# PRDM14 controls X-chromosomal and global epigenetic reprogramming of H3K27me3 in migrating mouse primordial germ cells

**DOI:** 10.1186/s13072-019-0284-7

**Published:** 2019-06-20

**Authors:** Anna Mallol, Maria Guirola, Bernhard Payer

**Affiliations:** 1 Centre for Genomic Regulation (CRG), The Barcelona Institute of Science and Technology, Dr. Aiguader 88, 08003 Barcelona, Spain; 2 Universitat Pompeu Fabra (UPF), Barcelona, Spain

**Keywords:** PRDM14, X-chromosome reactivation, PGCs, Epigenetic reprogramming, H3K27me3, Mouse

## Abstract

**Background:**

In order to prepare the genome for gametogenesis, primordial germ cells (PGCs) undergo extensive epigenetic reprogramming during migration toward the gonads in mammalian embryos. This includes changes on a genome-wide scale and additionally in females the remodeling of the inactive X-chromosome to enable X-chromosome reactivation (XCR). However, if global remodeling and X-chromosomal remodeling are related, how they occur in PGCs in vivo in relation to their migration progress and which factors are important are unknown.

**Results:**

Here we identify the germ cell determinant PR-domain containing protein 14 (PRDM14) as the first known factor that is instrumental for both global reprogramming and X-chromosomal reprogramming in migrating mouse PGCs. We find that global upregulation of the repressive histone H3 lysine 27 trimethylation (H3K27me3) mark is PRDM14 dosage dependent in PGCs of both sexes. When focusing on XCR, we observed that PRDM14 is required for removal of H3K27me3 from the inactive X-chromosome, which, in contrast to global upregulation, takes place progressively along the PGC migration path. Furthermore, we show that global and X-chromosomal reprogramming of H3K27me3 are functionally separable, despite their common regulation by PRDM14.

**Conclusions:**

In summary, here we provide new insight and spatiotemporal resolution to the progression and regulation of epigenome remodeling along mouse PGC migration in vivo and link epigenetic reprogramming to its developmental context.

**Electronic supplementary material:**

The online version of this article (10.1186/s13072-019-0284-7) contains supplementary material, which is available to authorized users.

## Background

The germ cell lineage has the unique function of transmitting genetic and epigenetic information from one generation to the next. In mice, germ cell development begins with the specification of PGCs, which then migrate through the hindgut in order to reach the genital ridges (future gonads), where they undergo meiosis and sex-specific differentiation into eggs and sperm. During migration and colonization of the gonads, the PGC epigenome is remodeled extensively at multiple levels, which includes global DNA-demethylation, erasure of genomic imprints, removal of histone H3 lysine 9 dimethylation (H3K9me2) and a global increase of the PRC2 (polycomb repressive complex 2)-associated H3K27me3 mark [1–7]. Around the same time, in female PGCs, the inactive X-chromosome is reactivated by XCR, which involves downregulation of the non-coding RNA Xist, the master regulator of X-inactivation, removal of repressive epigenetic marks like H3K27me3 and reactivation of X-linked genes [8–11]. Interestingly, H3K27me3 is therefore removed from the inactive X-chromosome, while it increases globally on autosomes. It is currently unknown, if these remodeling events are functionally linked or independent of each other.

XCR is a process linked to naïve pluripotency and germ cell fate [12, 13]. While XCR kinetics have been characterized during mouse and human germ cell development [8, 9, 11, 14, 15], the molecular mechanisms and factors, which are functionally important to reverse the inactive X-chromosome state, are largely unknown. Identifying such X-reactivation factors would be a key step toward revealing mechanistically how epigenetic memory can be erased in the germ line. We and others have shown that the transcriptional regulator PRDM14 plays a critical role in XCR in the mouse blastocyst epiblast in vivo and during induced pluripotent stem cell (iPSC) reprogramming [16] and epiblast stem cell (EpiSC) to embryonic stem cell (ESC) conversion [17] in vitro. In the absence of PRDM14, downregulation of Xist and removal of the H3K27me3 mark from the inactive X-chromosome are perturbed, likely due to repressive functions of PRDM14 on *Xist* and its activator *Rnf12/Rlim* [16, 18].

Besides being important for naïve pluripotency [19] and the associated XCR, PRDM14 is also critical for germ cell development. *Prdm14*-mutant mouse embryos show reduced PGC numbers [20], and overexpression of PRDM14 in epiblast-like cells (EpiLCs) is sufficient to induce germ cell fate in vitro [21]. Importantly, PRDM14 is a regulator of global epigenetic changes both in pluripotent stem cells and in PGCs [21, 22]. In particular, it has been shown that PRDM14 is responsible for the low global DNA-methylation levels characteristic of naïve pluripotent stem cells and PGCs, both by repressing DNA-methyltransferases and by recruiting DNA-demethylases of the TET family [19, 20, 23–26]. Besides controlling DNA-hypomethylation, PRDM14 is also important for global remodeling of histone marks. In migrating PGCs, *Prdm14*-mutant mouse embryos fail to downregulate the repressive H3K9me2 mark and its associated histone methyltransferase GLP/EHMT1 [20], most likely because *Glp/Ehmt1* is a directly repressed target gene of PRDM14 [27]. The PGC-specific upregulation of H3K27me3 on a global level is also impaired in *Prdm14*^−*/*−^ embryos [20] and genome-wide distribution of H3K27me3 is misregulated in *Prdm14*^−*/*−^ ESCs [19]. However, it is not known, whether these epigenetic defects also occur in *Prdm14*^+*/*−^ embryos.

Although the importance of PRDM14 for epigenetic remodeling in the germ cell lineage is known, how PRDM14 controls its different aspects in vivo is largely unresolved: For example, it remains unknown, if PRDM14 is important for X-chromosome reactivation in female PGCs, and if this is coordinated with genome-wide epigenetic changes occurring at that time. Furthermore, no information is available about the spatial distribution of germ cells along their migration path toward the gonads during epigenetic reprogramming. To address these questions, we here investigated epigenetic remodeling of the polycomb-associated H3K27me3 mark and its dependency on PRDM14 in migrating mouse PGCs. By using a whole mount embryo staining approach, we were able to collect spatiotemporal information about the epigenetic reprogramming process in mouse PGCs in relation to their migratory progress. We found that PRDM14 is critical both for X-chromosomal removal and for global upregulation of H3K27me3 in PGCs. We furthermore found differences in PRDM14-dosage dependence and kinetics of X-chromosomal and global H3K27me3 changes. While both events are controlled by PRDM14, they do not depend on each other and therefore must be regulated through different mechanisms. In summary, we describe here how PRDM14 regulates key aspects of epigenetic changes in germ cells and how this is linked with progression of germ cell migration and development.

## Results

### PRDM14 controls number and local distribution of PGCs during their migration

**Fig. 1:**
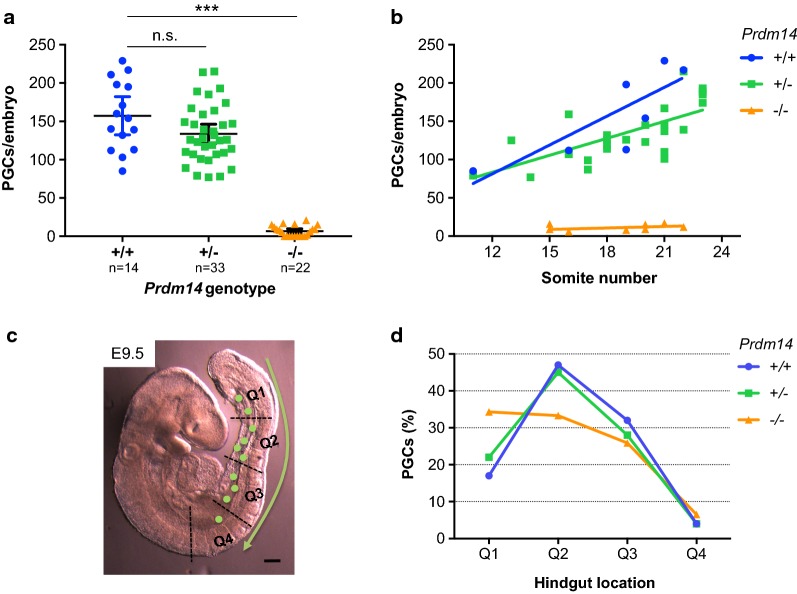
*Prdm14*^−*/*−^ E9.5 embryos have reduced PGC numbers with altered distribution along the hindgut. **a** Numbers of AP2γ-positive PGCs per embryo across *Prdm14* genotypes. Means and 95% confidence intervals are shown as error bars. Kruskal–Wallis test was used for statistical comparison (*p* < 0.001). **b** Linear regression of AP2γ-positive PGC numbers versus developmental stage (somite number). R-square values for *Prdm14*^+/+, +*/*−^ and ^−*/*−^ embryos were 0.659, 0.514 and 0.001, respectively. **c** Image of a E9.5 mouse embryo with PGCs (drawn green dots) migrating along the hindgut divided into four quadrants (Q1–Q4). Scale bar: 250 μm. **d** Distribution of PGCs along the hindgut across *Prdm14* genotypes

To address the function of PRDM14 for PGC number and migration, we immunostained whole mount embryonic day (E)9.5 mouse embryos from *Prdm14*^+*/*−^ x *Prdm14*^+*/*−^ heterozygous crosses for AP2γ, a specific marker and critical factor for PGC development [21, 28]. We found that migrating PGCs at this stage were spread throughout the hindgut and observed in *Prdm14*^−*/*−^ embryos a strong depletion of PGCs or even their absence (9/22 embryos), when compared to wild type and heterozygous littermates (Fig. 1a, Additional file 1: Fig. S1, Additional file 2: Table S1), which is consistent with a previous study of this mouse line [20]. Generally, PGC numbers increased with developmental progression (somite number) in *Prdm14*^+*/*+^ and *Prdm14*^+*/*−^embryos, while they remained low in *Prdm14*^−*/*−^ embryos (Fig. 1b). This is not due to a general developmental delay of *Prdm14*-mutant embryos at E9.5, as somite numbers were similar between different *Prdm14* genotypes (Additional file 1: Fig. S2a, Additional file 2: Table S1).

In order to analyze the distribution of PGCs in more detail, we divided the migration path along the hindgut into 4 quadrants (Fig. 1c), starting with quadrant 1 (Q1) at the posterior end at the base of the allantois and finishing with quadrant 4 (Q4) near the area where PGCs would exit the hindgut and enter the genital ridges (future gonads). At E9.5, PGCs would be found mostly in Q2 and Q3 in *Prdm14*^+*/*+^ and *Prdm14*^+*/*−^embryos, while PGCs in *Prdm14*^−*/*−^ embryos could be also frequently still found in Q1 (Fig. 1d, Additional file 1: Fig. S2b). This indicates that *Prdm14*-mutant PGCs might be slightly less efficient in initiating migration out of Q1, although they were also found in the other quadrants. In summary, *Prdm14*-mutant embryos show a strong reduction in total PGC numbers and some what reduced efficiency in PGC migration.

### PRDM14 is required for global upregulation of H3K27me3 in migrating PGCs

**Fig. 2:**
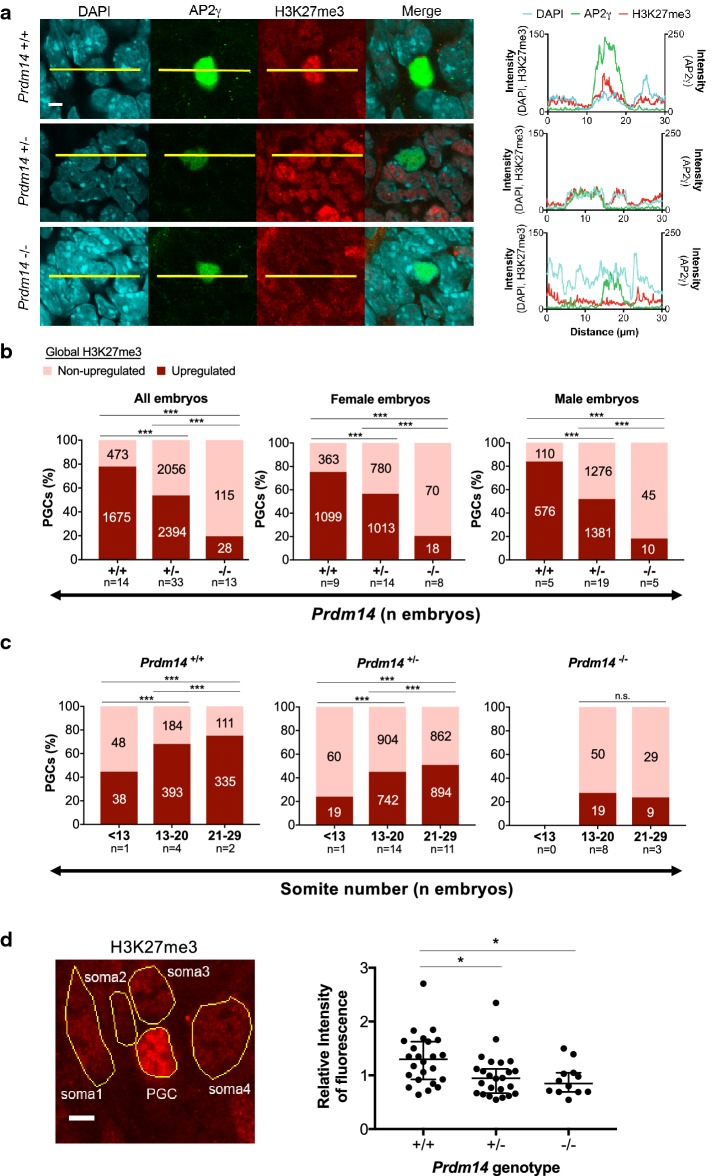
PRDM14 dosage controls global H3K27me3 levels in migrating PGCs in both sexes. **a** Representative images of AP2γ-positive PGCs with upregulated and non-upregulated global levels of H3K27me3 across different *Prdm14* genotypes. Scale bar: 5 µm. Intensity values for each channel along the yellow lines are plotted on the right. **b** Percentages of H3K27me3-upregulated and non-upregulated PGCs across *Prdm14* genotypes in male and female embryos. **c** Percentages of H3K27me3 upregulated and non-upregulated PGCs at different developmental stages (somite number). Labels in each column indicate number of PGCs analyzed. Chi-square test was used for statistical comparisons (*p* < 0.001). **d** Representative image of a PGC and four neighboring somatic cells (same group of cells as shown in **a** *Prdm14*^+*/*+^ above). Their areas were selected to quantify the mean intensity of H3K27me3 signal. Scale bar: 5 µm. Relative intensity of H3K27me3 staining in PGCs in comparison with neighboring somatic cells across *Prdm14* genotypes is plotted on the right. Medians and the 95% confidence intervals are shown as error bars. Kruskal–Wallis test was used for statistical comparison (*p* < 0.05)

As PRDM14 has a role in the global epigenetic reprogramming occurring in PGCs [20], we wanted to investigate in more detail its function for upregulating the H3K27me3 mark, which is a hallmark of migrating PGCs [4–6]. We therefore costained E9.5 embryos of different *Prdm14* genotypes with AP2γ and H3K27me3 antibodies (Fig. 2a, d). We scored H3K27me3 as being upregulated or non-upregulated in AP2γ-positive PGCs when compared to surrounding somatic cells. It thereby became apparent that H3K27me3 upregulation was in direct relation to PRDM14 dosage, as about 78% of wild-type PGCs had elevated H3K27me3 staining, while only 54% of heterozygote and only 20% of Prdm14-null PGCs did (Fig. 2b, d, Additional file 2: Table S1). These differences were observed to a similar degree both in male and in female embryos, indicating that PRDM14 controlled global H3K27me3 levels in a sex-independent manner.

When we assessed H3K27me3 upregulation in relation to developmental progression (somite number) at E9.5, we observed that H3K27me3 increased with somite number in *Prdm14*^+*/*+^ and *Prdm14*^+*/*−^, but not in *Prdm14*^−*/*−^ embryos (Fig. 2c). Interestingly, H3K27me3 levels did not seem to vastly change in PGCs within different quadrants of their migration path, indicating that global H3K27me3 upregulation depends more on overall developmental progression of the embryo than on the position of the PGCs along the hindgut (Additional file 1: Fig. S3). Overall, we conclude that global H3K27me3 upregulation in PGCs is dependent on PRDM14 dosage and progresses with developmental stage at equal rate in both sexes.

### X-chromosomal H3K27me3-erasure during XCR in female PGCs is dependent on PRDM14

**Fig. 3:**
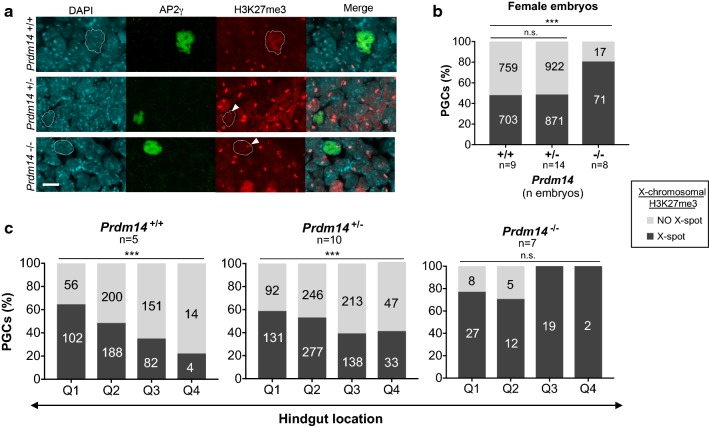
Erasure of H3K27me3-enrichment from the inactive X-chromosome in migrating female PGCs is dependent on PRDM14. **a** Representative images of AP2γ-positive PGCs with the presence or absence of H3K27me3 accumulation (H3K27me3 spot, white arrowheads) on the inactive X-chromosome. PGC nuclei are outlined in the H3K27me3 channel. Scale bar: 10 µm. **b** Percentage of H3K27me3 spot-positive and spot-negative PGCs across *Prdm14* genotypes. Labels in each column indicate number of cells analyzed. **c** Erasure of the H3K27me3-spot in PGCs of different *Prdm14* genotypes during their migration along the hindgut divided in four quadrants (Q1–Q4). Labels in each column indicate number of PGCs analyzed. Chi-square test was used for statistical comparisons (*p* < 0.001)

In contrast to the global increase of H3K27me3 stands the erasure of H3K27me3 from the inactive X-chromosome in female PGCs during the process of XCR [9, 11]. As these events occur around the same time and as XCR is controlled by PRDM14 in mouse blastocysts and during iPSC reprogramming [16], we wanted to know if PRDM14 is also required for XCR in PGCs. Thus, we scored the disappearance of the distinctive H3K27me3 accumulation from the inactive X-chromosome (“H3K27me3-spot”) in female PGCs in embryos of different *Prdm14* genotypes (Fig. 3a). While about half of both *Prdm14* wild-type (52%) and heterozygous (51%) PGCs have erased the H3K27me3-spot from the X, only around 19% of *Prdm14*-mutant PGCs have lost the spot at E9.5 (Fig. 3b, Additional file 2: Table S1). The erasure of the H3K27me3 spot occurred progressively in *Prdm14*^+*/*+^ and *Prdm14*^+*/*−^ PGCs during their migration along the hindgut, but did not progress with migration in *Prdm14*^−*/*−^ PGCs (Fig. 3c). In contrast to global H3K27me3 upregulation, removal of the X-chromosomal H3K27me3-spot therefore did not seem to be PRDM14-dose dependent and did not change substantially between E9.5 embryos of different somite number (Additional file 1: Fig. S4). Taken together, our results show that similar to its role in the pluripotent epiblast and during iPSC reprogramming [16], PRDM14 is also a key factor for XCR in PGCs by promoting the loss of the X-chromosomal H3K27me3 mark.

### X-chromosomal and global H3K27me3 reprogramming occur independently in female PGCs

**Fig. 4:**
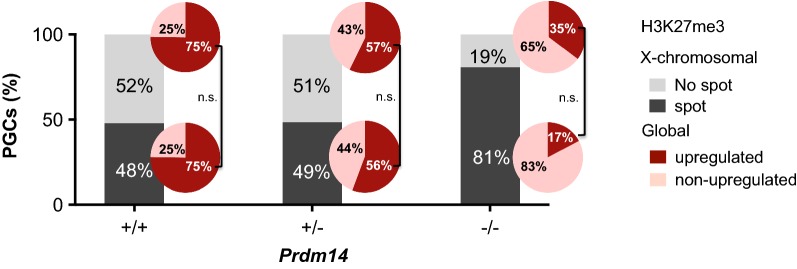
Changes in both X-chromosomal and global levels of H3K27me3 in female PGCs require PRDM14 but are independent of each other. Relationship between X-chromosomal (bar graphs, % of PGCs per genotype) and global (pie charts, % of PGCs per genotype within each H3K27me3 spot/no spot sub-category) levels of H3K27me3 in PGCs from female embryos of different *Prdm14* genotypes. Chi-square test was used to compare dependency of global of H3K27me3 upregulation on X-chromosomal removal for each *Prdm14* genotype (n.s., not significant; *Prdm14*^+*/*+^ *p* = 0.956, *Prdm14*^+*/*−^ *p* = 0.499, *Prdm14*^−*/*−^ *p* = 0.091)

**Fig. 5:**
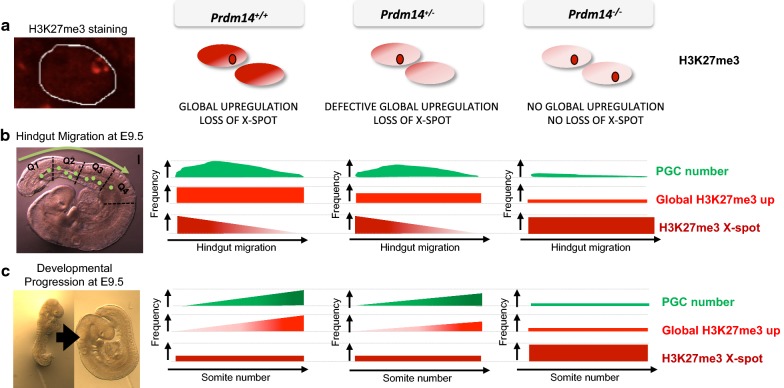
Summary of H3K27me3 dynamics in PGCs. **a** Schematic of H3K27me3 staining in representative female PGC nuclei of different *Prdm14* genotypes. **b** PGC numbers and H3K27me3 changes during PGC migration along the hindgut at E9.5. PGCs are most frequently seen in quadrant 2 (Q2), and their number is strongly reduced in *Prdm14*^−*/*−^ embryos. Global H3K27me3 levels are upregulated in most PGCs in *Prdm14*^+*/*+^ embryos throughout the hindgut, while it is less frequent in *Prdm14*^+*/*−^ and rarely seen in *Prdm14*^−*/*−^ embryos. The accumulation of H3K27me3 on the inactive X-chromosome (X-spot) is progressively lost during migration in both *Prdm14*^+*/*+^ and *Prdm14*^+*/*−^ PGCs, but mostly maintained in *Prdm14*^−*/*−^ PGCs. **c** PGC numbers and H3K27me3 changes in E9.5 embryos with different developmental progression (somite number). PGC numbers and global H3K27me3 levels increase with developmental progression in *Prdm14*^+*/*+^ and *Prdm14*^+*/*−^ (reduced upregulation), but not in *Prdm14*^−*/*−^ embryos. The X-chromosomal H3K27me3 spot is lost in both *Prdm14*^+*/*+^ and *Prdm14*^+*/*−^ PGCs independently of developmental progression, while *Prdm14*^−*/*−^ PGCs maintain the X-spot at all stages

H3K27me3 and its associated enzymatic complex, PRC2, are strongly enriched on the inactive X-chromosome in female cells. Therefore, we hypothesized, if the X-chromosome could act as a “sink” for PRC2, whereby global upregulation of H3K27me3 in PGCs would require first stripping PRC2 off the X-chromosome through XCR to make it available for acting elsewhere in the genome. In order to test this hypothesis, we investigated the relationship between X-chromosomal downregulation and global upregulation of H3K27me3 in female PGCs (Fig. 4, Additional file 1: Fig. S5). If the inactive X were acting as a sink for PRC2, we would expect global H3K27me3 not to be upregulated in PGCs harboring an X-spot. However, when comparing within the different genotypes the fraction of PGCs which have lost or retained the X-chromosomal H3K27me3-spot, we did not detect any significant differences in global H3K27me3 upregulation (Fig. 4). This indicates that losing the X-spot is not a general prerequisite for global H3K27me3 upregulation in female PGCs. Also the fact that *Prdm14*^+*/*+^ and *Prdm14*^+*/*−^ PGCs showed almost identical X-spot loss (52% in +/+ vs. 51% in +/−) but different global H3K27me3 upregulation levels (75% in +/+ vs. 56% in +/−) indicates that X-spot loss and global H3K27me3 upregulation are independent epigenetic events. In conclusion, while PRDM14 is both required for normal X-chromosomal and global H3K27me3 reprogramming in PGCs (Fig. 5), these events are mechanistically separable and do not depend on each other.

## Discussion

In this study, we have investigated the role of PRDM14 during epigenetic reprogramming in migrating mouse PGCs (summarized in Fig. 5). We thereby have uncovered multiple roles for PRDM14 during germ cell development. Most importantly, we have identified PRDM14 as the first factor with functional importance for XCR in the germ cell lineage. In particular, we found that erasure of H3K27me3 mark from the inactive X-chromosome, a key step during X-reactivation [9, 11, 29, 30], occurred progressively along the migration path of female PGCs and required PRDM14. This shows that PRDM14 has a universal role during X-reactivation whenever it is expressed; in the mouse blastocyst, during iPSC reprogramming [16] and in the germ cell lineage (this study).

Second, we uncovered that global H3K27me3 upregulation in PGCs is dependent on PRDM14 in a dosage-sensitive manner, as *Prdm14*^+*/*−^ and *Prdm14*^−*/*−^ PGCs showed a progressive defect. Interestingly, we observed that H3K27me3 upregulation seemed to mostly depend on the developmental stage of the embryo rather than the PGC position along the migratory path, which is in contrast to the kinetics of X-specific H3K27me3 removal.

Another key finding from our study is that the global upregulation and X-chromosomal depletion of the H3K27me3 mark seem to be controlled by distinct mechanisms despite their common dependency on PRDM14. This is based on several observations: First, global H3K27me3 upregulation is sensitive to PRDM14 dosage, while X-specific H3K27me3 removal is not. Second, global upregulation correlates with embryonic stage (somite number), while X-chromosomal H3K27me3 loss occurs progressively during PGC migration. Third, global H3K27me3 upregulation occurs with similar kinetics in male and female embryos and is affected to a similar extent by loss of PRDM14 in both sexes. Finally, X-chromosomal depletion of H3K27me3 does not seem to be required for global H3K27me3 upregulation on a single-cell level.

How then could PRDM14 control H3K27me3 remodeling on autosomes versus on the X-chromosome? On the global scale, previous studies suggested that PRDM14 could directly interact with the PRC2 complex in pluripotent stem cells and thereby facilitate its recruitment to PRDM14 target genes [19, 31] or that PRDM14 could activate expression of the PRC2 component *Suz12* [24]. More recent studies, however, challenged this view and proposed that PRDM14 mainly acts though its binding partner and co-repressor CBFA2T2/MTGR1 [27, 32, 33], suggesting that PRC2 might be recruited secondarily to PRDM14 targets. Furthermore, PRDM14 is required for the low DNA-methylation levels in PGCs [24, 34]. Interestingly, PGC-like cells cultured in vitro lose DNA-methylation, while compensating by upregulating promoter H3K27me3 levels to ensure repression [34, 35], with BLIMP1 being a key factor in redistributing the H3K27me3 mark [36]. Future studies will need to address, if PRDM14 regulates global H3K27me3 upregulation in PGCs through direct or indirect mechanisms.

Regarding its X-chromosome-specific role, we have previously shown that PRDM14 binds to regulatory regions at *Xist* intron 1 and upstream of the *Xist*-activator *Rnf12* [16] and thereby facilitates repression of *Xist* during XCR. As H3K27me3 accumulation on the X-chromosome is *Xist*-dependent [37–40], PRDM14 might thereby regulate X-chromosomal H3K27me3-depletion by downregulating *Xist*. The fact that H3K27me3 removal from the X-chromosome occurs progressively in PGCs along their migration path could speak in favor of a cell proliferation and replication dependent, passive dilution mechanism, similar as it has been proposed for global DNA-demethylation in PGCs [41, 42]. Nevertheless, also active mechanisms could play a role like removal of the mark by the H3K27me3-demethylase KDM6A/UTX, which has a partial effect on H3K27me3-demethylation during XCR in mouse blastocysts [30].

Finally, we have confirmed that *Prdm14*^−*/*−^ embryos displayed severely compromised germ cell numbers [20] and observed for the first time their reduced efficiency in PGC migration. This is in line with a recent study [43], which found that genes implicated in cell migration are bound by PRDM14 in PGC-like cells derived in vitro, suggesting that PRDM14 might be potentially involved in controlling PGC migration.

## Conclusions

In summary, here we have provided a first spatial roadmap of autosomal and X-chromosomal reprogramming of H3K27me3 during mouse PGC migration in vivo. We showed that X-chromosomal and global reprogramming occur independently of each other, but that they rely both on the key germ cell factor PRDM14. By adding to our understanding of the epigenetic reprogramming required for germ cell development in vivo, we provide a framework for assessing and improving the quality of in vitro-derived gametes.

## Methods

### Embryo isolation

Mouse care and procedures were conducted according to the protocols approved by the Ethics Committee on Animal Research of the Parc de Recerca Biomèdica de Barcelona (PRBB) and by the Departament de Territori i Sostenibilitat of the Generalitat de Catalunya.

*Prdm14* mutant mice [20] were maintained in a predominant C57BL/6 strain background. *Prdm14* heterozygous mice were mated, and resulting embryos were harvested from pregnant females at E9.5 and dissected from maternal tissues.

### Whole mount immunostaining

The whole mount embryo immunostaining was performed as described in [20]. The primary antibodies used were rabbit polyclonal anti-AP2γ (TCFAP2C) (Santa Cruz Biotechnology sc-8977) and mouse monoclonal anti-H3K27me3 (Active Motif 61707, clone MABI0323). The secondary antibodies used were donkey anti-rabbit IgGAlexa Fluor 488 and goat anti-mouse IgGAlexa Fluor 555 (Molecular Probes A21206 and A21424).

After the immunostaining, the somite number of every embryo was counted under the stereomicroscope. Then the embryo was split in two parts: the head was used for genotyping, and the hindgut was dissected and mounted in Vectashield (Vector Laboratories) for observation with confocal microscopy.

### Image capture and analysis

Bright-field images of the whole embryos were captured on a stereomicroscope using the Leica application suite software (Leica Microsystems, Wetzlar, Germany). Fluorescence imaging of the hindguts was performed on an inverted Leica TCS SP5 confocal microscope using the Leica Application Suite Advanced Fluorescence software. Z-stack images (1.5 μm intervals) of the full hindgut were acquired. Color setting and image processing were performed in Fiji [44] and Volocity (PerkinElmer) software.

The total length of the hindgut was divided in four quadrants (Q1–Q4 from the tail tip to the genital ridges, Fig. 1c) to ease the analysis of the location of AP2γ-positive PGCs. The global nuclear intensity of H3K27me3 staining and the presence of the X-spot were analyzed by visually inspecting all images from the confocal z-stack where a particular PGC was present in Volocity software. PGC showing a lower or similar nuclear H3K27me3 staining to the neighboring somatic cells in the same region/focal planes were categorized as “non-upregulated.” PGCs showing H3K27me3 nuclear staining levels more intense than the majority of neighboring somatic cells were categorized as “upregulated.” Image analysis was performed before *Prdm14* genotyping to avoid bias. Alternatively, to quantitatively compare H3K27me3 staining levels of PGCs relative to somatic neighbors, a subset of PGCs from every *Prdm14* genotype was analyzed by pixel intensity quantification with Fiji [44]. The median of the H3K27me3 average signal from four neighboring somatic cells was calculated and set as 1, and the H3K27me3 average signal from the PGC relative to this value was indicated (Fig. 2d).

### *Prdm14* and sex genotyping

*Prdm14* genotype was determined by polymerase chain reaction after image analysis. Primer sequences are detailed in [20].

The sex of the embryo was determined by the presence of the H3K27me3 spot corresponding to the silent X-chromosome in somatic cells of female embryos [38].

### Statistical analysis

Several E9.5 litters were collected, and the results from all embryos were pooled and analyzed with IBM SPSS and GraphPad Prism Statistics software. For qualitative variables Chi-square test was used, and for quantitative variables, the nonparametric Kruskal–Wallis test was used. Pairwise comparisons with Bonferroni’s correction were performed using the Mann–Whitney *U* test. For lineal regression modeling, *R*^2^ coefficient was calculated. In all cases, *p*  <  0.05 was considered statistically significant.

## Additional files

**Additional file 1: Figures S1–S5.** Figure S1 (related to Fig. 1). Migrating PGCs in E9.5 mouse embryos of different *Prdm14* genotypes. Figure S2 (related to Fig. 1). *Prdm14* genotype, developmental progression and PGC distribution during migration. Figure S3 (related to Fig. 2). Summary of global H3K27me3 levels in PGCs across somite number, migration progression and *Prdm14* genotypes. Figure S4 (related to Fig. 3). Summary of the removal of X-chromosomal H3K27me3 accumulation (spot) in PGCs across somite number, migration progression and *Prdm14* genotypes. Figure S5 (related to Fig. 4). Distribution of PGCs with different global and X-chromosomal H3K27me3 status dependent on *Prdm14* genotype.

**Additional file 2: Table S1.** Summary table of embryos and PGCs analyzed in this study.

## Abbreviations

EpiLC: epiblast-like cell
ESC: embryonic stem cell
H3K27me3: histone H3 lysine 27 trimethylation
iPSC: induced pluripotent stem cell
PGC: primordial germ cell
PRC2: polycomb repressive complex 2
PRDM14: PR-domain containing protein 14
XCR: X-chromosome reactivation
Xist: X-inactive specific transcript

## Availability of data and materials

All data generated or analyzed during this study are included in this published article and its supplementary information or are available from the corresponding author on reasonable request.
