# Supplementary material for: PRDM14 controls X-chromosomal and global epigenetic reprogramming of H3K27me3 in migrating mouse primordial germ cells
Source: Epigenetics Chromatin. 2019 Jun 20;12:38. doi: 10.1186/s13072-019-0284-7 (PMC6585054; doi:10.1186/s13072-019-0284-7)
Supplement: Supplementary file 1 — Additional file 1: Figures S1–S5. Figure S1 (related to Fig. 1). Migrating PGCs in E9.5 mouse embryos of different Prdm14 genotypes. Figure S2 (related to Fig. 1). Prdm14 genotype, developmental progression and PGC distribution during migration. Figure S3 (related to Fig. 2). Summary of global H3K27me3 levels in PGCs across somite number, migration progression and Prdm14 genotypes. Figure S4 (related to Fig. 3). Summary of the removal of X-chromosomal H3K27me3 accumulation (spot) in PGCs across somite number, migration progression and Prdm14 genotypes. Figure S5 (related to Fig. 4). Distribution of PGCs with different global and X-chromosomal H3K27me3 status dependent on Prdm14 genotype. [file 13072_2019_284_MOESM1_ESM.pdf]

SUPPLEMENTARY INFORMATION

Supplementary Figures

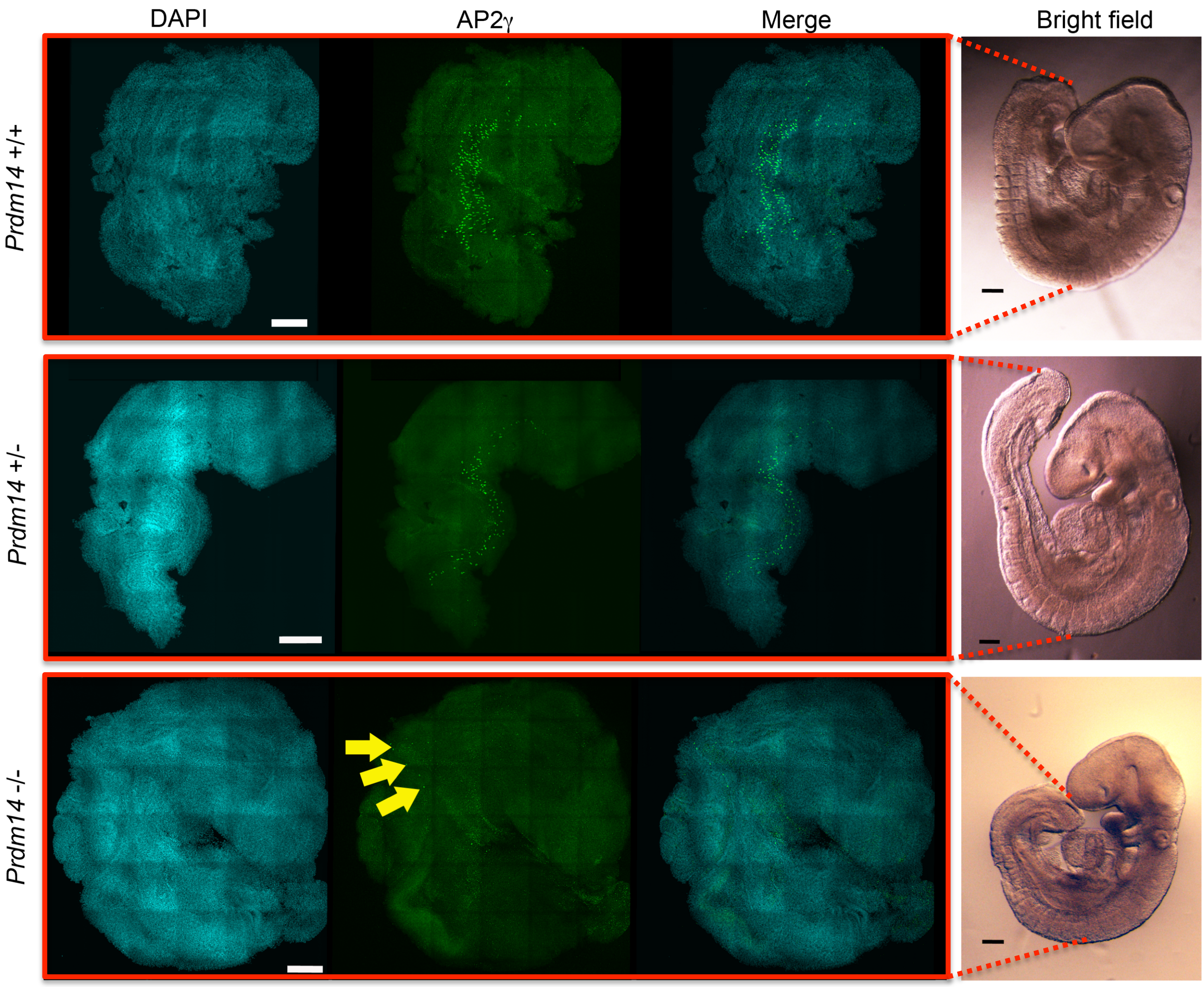

Figure S1 (related to Fig. 1). Migrating PGCs in E9.5 mouse embryos of different *Prdm14* genotypes. Representative immunofluorescence images of hindgut regions (left) and corresponding bright field images of E9.5 embryos (18-19 somites, right) after whole mount antibody staining. Migrating PGCs express AP2γ. Arrows indicate four PGCs in a *Prdm14*$^{-/-}$ hindgut. Scale bars: 250 μm.

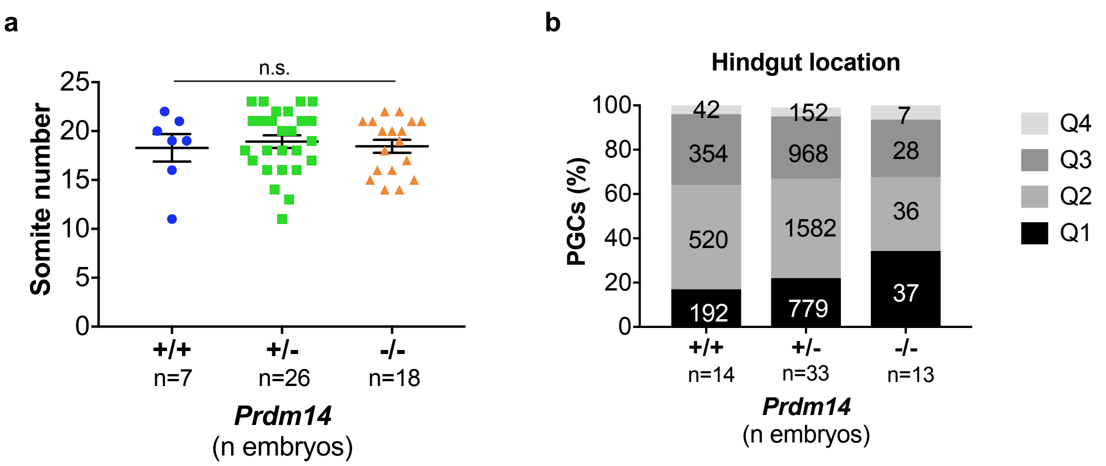

Figure S2 (related to Fig. 1). *Prdm14* genotype, developmental progression and PGC distribution during migration. **a** The *Prdm14* genotype does not affect developmental progression in E9.5 embryos (Kruskal-Wallis test, p=0.853). Somite numbers per embryo are plotted as marker of developmental progression versus *Prdm14* genotype. Mean somite number and standard error (error bars) are given for each genotype. **b** Distribution of PGCs along the hindgut across *Prdm14* genotypes. Labels in each category indicate numbers of PGCs counted.

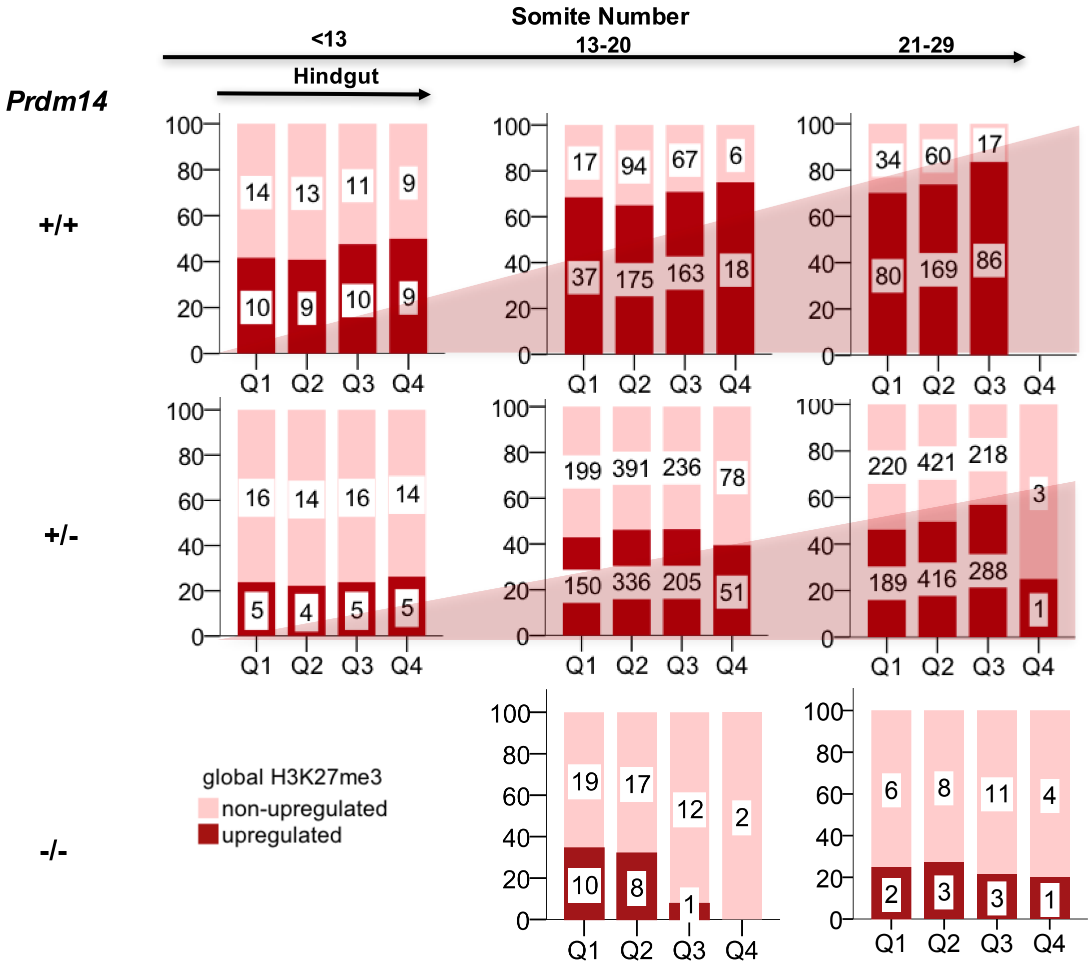

Figure S3 (related to Fig. 2). Summary of global H3K27me3 levels in PGCs across somite number, migration progression and *Prdm14* genotypes. The pink triangles symbolize the trend of increasing frequencies of PGCs with high global H3K27me3 levels with developmental progression (increasing somite number) in *Prdm14*$^{+/+}$ and *Prdm14*$^{+/-}$ (to a lesser degree than in *Prdm14*$^{+/+}$), but not in *Prdm14*$^{-/-}$ embryos.

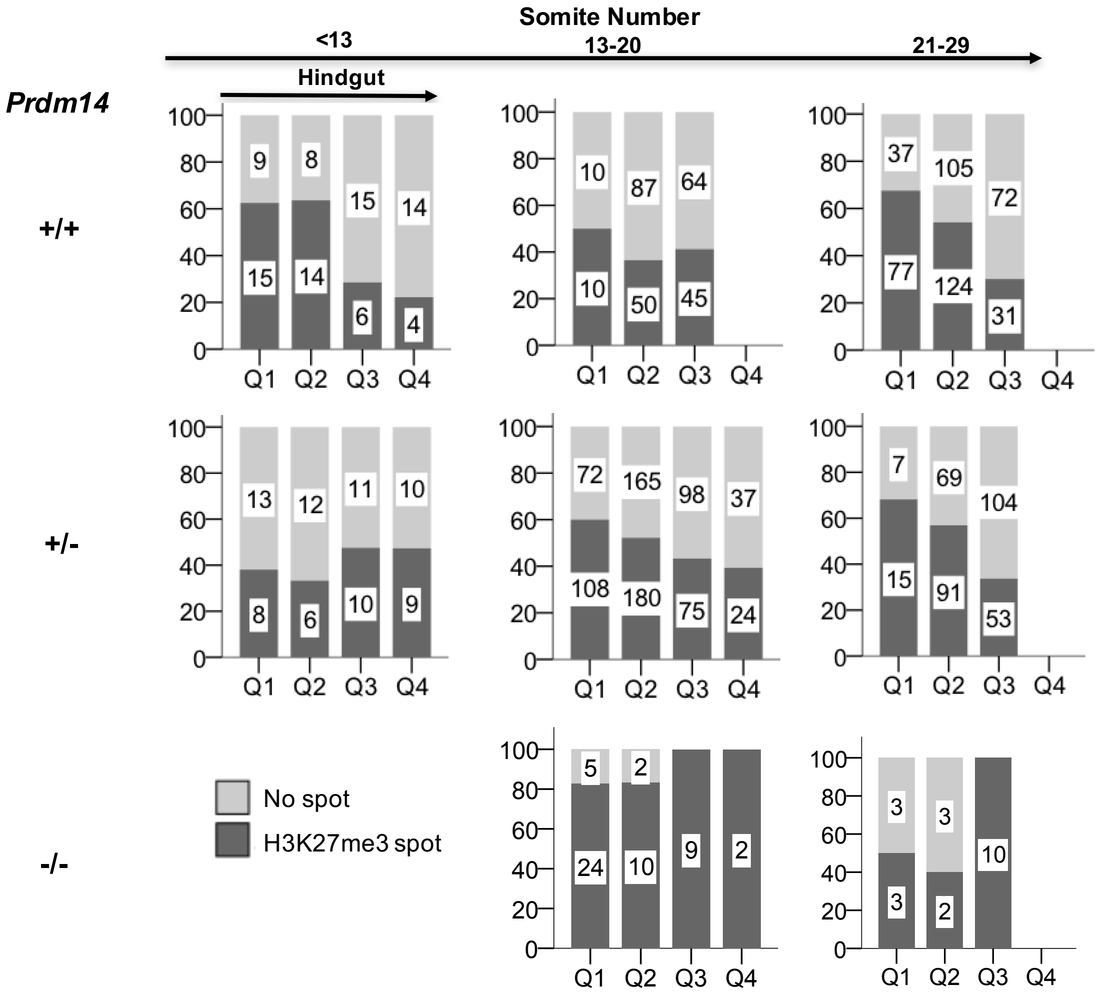

Figure S4 (related to Fig. 3). Summary of the removal of X-chromosomal H3K27me3 accumulation (spot) in PGCs across somite number, migration progression and *Prdm14* genotypes. A trend in removal of the H3K27me3 spot can be observed with progressing migration of PGCs in *Prdm14*$^{+/+}$ and *Prdm14*$^{+/-}$, but not in *Prdm14*$^{-/-}$ embryos.

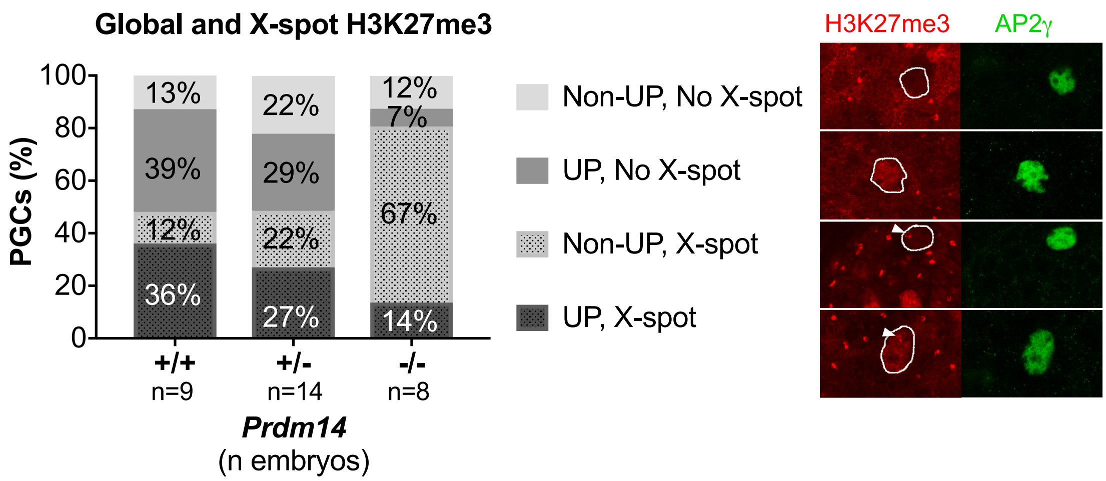

Figure S5 (related to Fig. 4). Distribution of PGCs with different global and X-chromosomal H3K27me3 status dependent on *Prdm14* genotype.

The bar chart shows the frequency of AP2γ-positive PGCs of the different H3K27me3 categories depicted in the immunostaining images on the right, in relation to *Prdm14* genotype. UP, global upregulated H3K27me3 staining in relation to surrounding somatic cells; X-spot, X-chromosomal H3K27me3 accumulation (white arrowheads).
