# Supplementary material for: PRDM14 controls X-chromosomal and global epigenetic reprogramming of H3K27me3 in migrating mouse primordial germ cells
Source: Epigenetics Chromatin. 2019 Jun 20;12:38. doi: 10.1186/s13072-019-0284-7 (PMC6585054; doi:10.1186/s13072-019-0284-7)
Supplement: Supplementary file 2 — Additional file 2: Table S1. Summary table of embryos and PGCs analyzed in this study. [file 13072_2019_284_MOESM2_ESM.docx]

**Supplementary Table S1**

| Genotype | N Embryos  (male/female) | Somite number  (mean ± SD) | PGC number  (mean ± SD) | % Global H3K27me3 UP  (mean ± SD) | % loss of H3K27me3 X-spot  (mean ± SD) |
| --- | --- | --- | --- | --- | --- |
| *Prdm14* +/+ | 14 (5/9) | 18.29 ± 3.73^a^ | 153.43 ± 44.01^a^ | 76.26 ± 17.38^a^ | 52.91 ± 8.58^a^ |
| *Prdm14* +/- | 33 (19/14) | 18.92 ± 3.27^a^ | 134.85 ± 37.89^a^ | 52.30 ± 18.57^b^ | 50.61 ± 7.51^a^ |
| *Prdm14* -/- | 22 (10/12) | 18.44 ± 2.83^a^ | 7.20 ± 6.94^b^ | 25.58 ± 25.69^c^ | 15.87 ± 17.68^b^ |

^a,b,c^ Values with different superscripts differ significantly from each other within the same column in the Kruskal-Wallis test (P<0.05).

Means and standard deviations (SD) in each column have been calculated per embryo.
